# Change in Denture Procedures after Implementation of National Health Insurance Coverage for the Elderly in Korea: A Nationwide Database

**DOI:** 10.3390/ijerph18052283

**Published:** 2021-02-25

**Authors:** Ye Seol Lee, Juyeong Kim, Yoon Soo Choy, Eunkyong Kim, Jaehyun Yoo

**Affiliations:** 1 Department of Rehabilitation Medicine, Seoul National University Hospital, Seoul National University College of Medicine, Seoul 03080, Korea; yeseol.lee127@gmail.com; 2 National Traffic Injury Rehabilitation Research Institute, National Traffic Injury Rehabilitation Hospital, Yangpyeong-eup 12564, Korea; 3 Department of Public Health, Sahmyook University, Seoul 01795, Korea; jennajyk394@gmail.com; 4 Center for Global Healthcare, Eulji University, Seongnam 13135, Korea; yschoy@eulji.ac.kr; 5 Laboratory of Health & Exercise Science, Sahmyook University, Seoul 01795, Korea; kimitey@naver.com; 6 Department of Leisure and Sports Studies, Sahmyook University, Seoul 01795, Korea

**Keywords:** denture, elderly, policy implementation

## Abstract

Background: Dental health is an important factor in daily life routines and is closely associated with maintaining a health-related quality of life. This study examined denture procedure changes after implementation of the National Health Insurance (NHI) Coverage of Dentures for the elderly. Methods: We used the “Korean Community Health Survey (KCHS)” developed by the Korea Centers for Diseases Control and Prevention. We analyzed the association between policy implementation and dental health-related outcomes using difference-in-differences (DID) analysis to compare patients aged ≥75 with those 65–74 years before and after coverage. Results: A comparison of age groups and coverage periods showed that patients aged ≥75 years had higher (OR: 1.038, 95% CI: 1.021–1.055) procedure rates after coverage. In particular, elderly patients on medical aid had significantly higher denture procedure rates, while those without oral health screening were more likely to have denture procedures. Conclusions: This study determined the impact of the NHI Coverage of Denture procedure policy for the elderly and found increased denture treatments in the elderly. This policy appeared to positively affect older patients by increasing denture procedures for low-income and medical aid beneficiaries. Hence, the government needs to increase oral health examination and dental health policies for the elderly.

## 1. Introduction

Currently, aging of the population has resulted in an increased incidence of health-related problems in the elderly, such as declining physical function and other health disorders. Older individuals often face financial difficulties owing to their insufficient incomes, because they do not engage in any economic activity. Furthermore, the declining birth and mortality rates have increased the elderly population of South Korea and some other developed countries, which have become “aged societies.” Therefore, the Korean government has implemented numerous policies to alleviate the economic, social, and mental health problems of the elderly.

There are numerous health policies aimed at providing the “right to health” to the elderly. In Korea, medical health policies targeting the elderly are divided into categories, including elderly policy, elderly support, and long-term care insurance [1]. The elderly policy covers services such as health and welfare for older individuals, services related to dementia, prevention of blindness, and taking care of older people living alone. Elderly support is targeted at improving welfare and social amenities for the elderly. Long-term care insurance is a social insurance that offers support services for physical activity and daily life for those who find it difficult to perform the usual daily activities because of their advanced age and senility. These policies are intended to provide support for the elderly to encourage a healthy lifestyle without restrictions in daily life.

Dental health is important for daily life and is closely related to maintaining a healthy quality of life [2]. Richmond et al. found that poor dental health is negatively associated with general health conditions [3]. Another study reported that dissatisfaction with chewing ability had a significant effect on the quality of life by depriving individuals of the pleasure of eating what they desired [4]. Therefore, the National Health Insurance (NHI) initiated coverage of dentures for the elderly in Korea in July 2012. The insurance aims to promote health and quality of life by improving the chewing ability of elderly patients. Both complete and partial dentures are included in the coverage of the denture policy, and its scope was expanded from individuals aged ≥75 years in July 2012 to older people ≥70 years in July 2015, and further to older people ≥65 years from July 2016 [5]. The rates of out-of-pocket expenditure are as follows: NHI subscribers pay 50% of the total denture expenditure, and in the lower income group, older individuals with rare diseases pay 20% of the cost, while elderly patients with chronic diseases pay 30% of the out-of-pocket cost. Furthermore, Type 1 patients who are medical aid beneficiaries pay 20% of the total expenditure, while Type 2 patients pay 30% of their dental costs. 

In fact, the percentage of the denture-wearing patient population registered in the NHI has been reported to have increased at an average annual rate of 82.8% [6]. However, these results were based on studies targeting older patients with dentures, who were covered under the NHI denture policy. Therefore, the purpose of this study was to determine the potential impact of the coverage of dentures for the elderly under the NHI policy by analyzing the change in denture procedures for the elderly following the implementation of the policy.

## 2. Material and Methods

### 2.1. Data Resource

In Korea, the coverage of dentures for the elderly under NHI commenced in July 2012. To compare the procedures before and after this policy was implemented, cross-sectional data from 2011 and 2013 from the Korean Community Health Survey (KCHS) developed by the Korea Centers for Diseases Control and Prevention (KCDC) were used. The KCHS is an annual nationwide survey at the level of cities, counties, and districts targeting South Koreans aged ≥19 years. The KCHS has been conducted annually in every administrative district since 2008 to provide information on local residents’ health behaviors and the use of medical services within the country [7].

Multistage stratified random sampling is used to select representative households in 253 local districts. South Korea consists of eight provinces, one special autonomous province, six metropolitan cities, and one special city, which are further divided into 253 administrative districts, such as cities, counties, and districts. Samples for the KCHS are selected in proportion to the sampling probability for each of the 253 districts, based on the number of households and the sample size. Therefore, these data are representative of the national population [7]. 

The questionnaire for the KCHS has been cooperatively developed by a working group of the health indicator standardization subcommittee, the KCDC staff, and public health officials from 16 metropolitan cities and provinces with 253 regional sites [7]. The KCDC conducts quality control tests and retest procedures for the reliability of the responses to the KCHS questionnaires [8]. The KCHS is conducted using face-to-face, computer-assisted personal interviews. Well-trained interviewers visit and interview each sampled household in 253 districts. Interviewers read questions to respondents from computer screens. Data are collected from August to October every year to promote comparisons by year between the results [7]. 

A total of 229,226 and 228,781 individuals aged ≥19 years were included in 2011 and 2013, respectively. Of these, 17,264 elderly individuals aged ≥65 years were asked to respond to surveys about variables related to dental health for the purpose of this study.

### 2.2. Study Variables

For the survey data, the dependent variable, that is, the status of denture procedures, had binary outcomes of having dentures or not having dentures. The data were considered based on the denture policy, and we divided the participants into age groups of 65–74 years and ≥75 years. In addition, we divided the investigation period into before and after coverage (2011 and 2013, respectively). The independent variables were dental health and individual characteristics. The dental-health-related variables were missing teeth (yes/no), perceived dental health status (good, not bad, and bad), undergoing oral health examinations (yes/no), and periodontal disease. With regards to periodontal disease, we divided the patients into the following three groups: no disease, presence of at least one disease, and data not available. The variables for the individual characteristics were sex, region, economic activity, family income, health insurance coverage, education, marital status, smoking, and drinking. Variables for family income, such as salary, property income, pensions, interest, subsidies, and allowances for one year, were presented as quartiles of total income and data not available.

### 2.3. Statistical Analysis

For all categorical variables, we used chi-square tests to calculate the distribution of characteristics according to age and policy change. To analyze the association between policy implementation and outcomes, we used a multivariate difference-in-differences (DID) regression analysis [9]. The DID analysis was used to reduce the probabilities of time-invariant omitted variables in the analysis of time trends by comparing the results before and after the policy reform [10,11]. This analysis enabled the comparison of elderly patients aged ≥75 years with those aged 65–74 years before and after coverage. We used multivariable logistic regression to confirm the association between having denture and other dental health-related or socioeconomic characteristics. Differences were considered statistically significant at *p* < 0.05, and the SAS program (ver. 9.4, SAS Institute, Cary, NC, USA) was used for all calculations and analyses.

## 3. Results

Table 1 shows the dental-health-related characteristics of the study group according to age and denture procedure before and after coverage initiation. Before coverage, the denture procedure rates in the missing teeth group were 71.3% and 51.6% in the 65–74- and ≥75-year-old individuals, respectively, while after coverage, the rates were 70.4% and 57.1%, respectively. In the group that received oral health screening, the denture procedure rates based on age groups (65–74 years and ≥75 years were 52.8% and 50.4% in 2011 and 51.3% and 49.5% in 2013, respectively. For the NHI subscribers aged 65–74 years and ≥75 years, the denture procedure rates were 47.1% and 40.3% before coverage and 48.7% and 43.8% after coverage, respectively. Furthermore, the 65–74- and ≥75-year-old individuals receiving medical aid showed denture procedure rates of 38.7% and 32.4% in 2011 and 35.5% and 38.9% in 2013, respectively (Supplementary Materials, Table S1).

**Figure 1:**
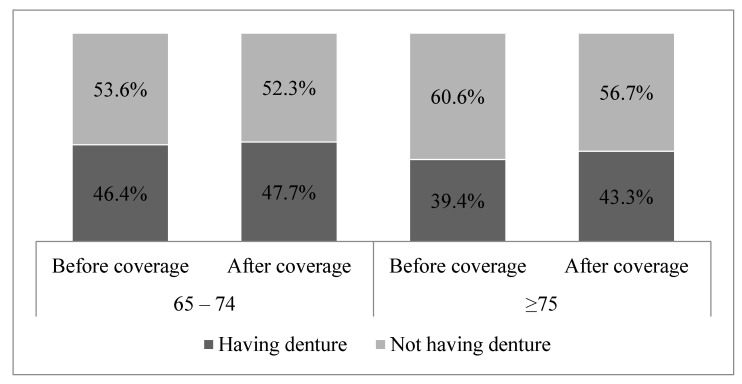
Change in denture procedure for the elderly before and after coverage (*p* < 0.0001).

Figure 1 shows the graph of the change in denture procedure rate in the elderly before and after coverage initiation, which reveals that the rates increased after coverage. In particular, the rate in the target group of the policy, the elderly aged ≥75 years, has increased more than that in the other group.

We performed a logistic regression analysis to examine the association between denture procedures and various characteristics, and the results are shown in Table 2. In elderly individuals aged ≥75 years, the dental procedure rates before coverage (odds ratio (OR) 0.875 and 95% confidence interval (CI) 0.859–0.890) were lower than those after coverage (OR 1.060, 95% CI 1.043–1.078). In particular, the DID analysis, which compared age groups before and after coverage, revealed that in elderly individuals aged ≥75 years, the procedure rate after coverage (OR 1.038, 95% CI 1.021–1.055) were higher than those before coverage. Analysis of the dental characteristics revealed that elderly individuals with missing teeth had more denture procedures (OR 1.969, 95% CI 1.934–2.004) and the group that received oral health examinations had a significantly higher rate of denture procedures than the unexamined group (OR 1.119, 95% CI 1.091–1.148). 

The group in which individuals perceived their oral health status as “not bad” was subjected to more denture procedures (OR 1.251, 95% CI 1.189–1.317) than the other groups. However, the group in which individuals perceived their oral status as “bad” was more likely to not have denture procedures (OR 0.727, 95% CI 0.696–0.760). Furthermore, we found that the group with periodontal disease had more denture procedures than those without periodontal disease. The analysis of the general characteristics of patients undergoing denture procedures revealed that there were fewer men than women, and older individuals living in the city were more than those living in rural areas. The group that engaged in economic activity was more likely to undergo denture procedures than the group that did not, but family income variables did not appear to have an effect on denture procedure rates. The medical aid group had significantly lower denture procedure rates than the NHI subscribers’ group. 

Table 3 shows the results of the DID analysis comparing the target elderly before and after coverage, focusing on health insurance coverage, oral health examinations, and family incomes. Elderly individuals ≥75 years old with both the NHI policy and medical aid, were more likely to have dentures after coverage than before. In particular, elderly individuals with medical aid had significantly higher denture procedure rates than those with NHI. The group not undergoing oral health screening was more likely to undergo denture procedures than those who had regular screenings, but the opposite situation was not statistically significant. Finally, the low family income group had higher denture procedure rates than those of the high family income group.

## 4. Discussion

The NHI policy aims to provide equal medical service to all people and guarantees their “right to health.” Numerous countries have achieved the goal of the NHI plan by addressing health disparities through sustained efforts to improve healthcare accessibility. In particular, a dental health policy is critical to daily health maintenance and not just preventing the pain of dental diseases [12]. In this study, we obtained several insights into the change in denture procedures after the implementation of the coverage of dentures for the elderly under NHI. First, there was an increase in denture procedures in medical aid beneficiaries and in the low-income group. In general, studies examining the association between income and visiting a dentist have revealed that low-income groups are not likely to visit a dentist [2,13]. Compared with other diseases, the importance of visiting a dentist, which is associated more with the convenience of living than it is with other diseases, is relatively downplayed because it is a burden for a low income household [14]. In addition, there is the possibility of limited use of dental health care [15,16]. 

The poverty rate of elderly Koreans is reported to be extremely high, and the income of older individuals is lower than the average income of individuals in the Organization for Economic Co-operation and Development countries [17]. The employment rate of the elderly is high, with the highest proportion (28.9%) working as day laborers or in primary industries [18]. The incomes of the elderly are low, and therefore the economic burden on household budgets in elderly Koreans is very high [19,20]. Older Korean individuals spend three times more on medical expenditures than other age groups, and show a high prevalence of chronic diseases [21,22]. The burden of medical expenditure is highest among the households of elderly Koreans who frequently visit the hospital [19]. The problems associated with the elderly have become a social issue, considering that Korea is rapidly becoming an aged society.

Therefore, the NHI denture coverage for the elderly was initiated to promote quality of life by reducing the inconvenience of impaired chewing ability and the financial burden of seniors, by having the government pay half the cost of denture treatments [5]. When policies providing health expenditure are initiated, healthcare access for low-income groups is increased. In particular, to resolve the unmet need to visit the dentist, medical expense support is necessary for low-income groups [2,14,15,23]. The results of this study show that denture procedures for the elderly target group of the policy and, particularly, those with low income or medical aid benefits, was fortunately increased, which confirms the positive impact of the policy. Although a moral hazard may occur when medical expenditure is increased rapidly by the provision of medical expense support, it has a positive effect because the policy provides the same medical service to the elderly that other individuals receive, without depending on their economic strength, and thereby satisfies their medical needs.

Second, denture procedures in elderly individuals not receiving oral health screening were higher than those in patients who were receiving screenings. This result can be interpreted to mean that elderly individuals who no longer required to be concerned about their dental treatment costs underwent more denture procedures. In Korea, the national oral health examination, which is conducted once every 2 years, targets people aged ≥40 years, except for infants. Furthermore, considering that oral health examinations are included in health screenings, Korean people have several opportunities to receive dental check-ups. Nevertheless, the rate of elderly individuals aged ≥65 years undergoing oral health examination was remarkably low at 17.8% [24]. This observation indicates a lack of policies for promoting dental-health-related services in Korea. Unlike treatments for other diseases, those for dental disorders may not adequately restore teeth to their original state of health once they are damaged [23]. Regular dental examinations can prevent damage to teeth, thereby, reducing the inconvenience that would be experienced following tooth damage. Furthermore, because people can accurately determine their state of dental health, it is possible to reduce unnecessary denture treatments and excessive treatment costs [2,25]. Therefore, by promoting oral health screening for the elderly, as well as examinations related to chronic diseases, the government is attempting to develop a steady interest in the dental policies targeting the elderly [2,26]. Numerous studies have reported that dental hygiene training or an interest in oral care contributes to preventing dental diseases, such as periodontal diseases [27,28]. Therefore, people with considerable interest in maintaining their dental health do not have to undergo denture procedures. We hope that this study will contribute to providing a strategy to encourage people to have a steady interest in such health policies. In addition, the government should consider promoting an overall health policy to increase the interest of the population and improve the quality of general as well as oral health examinations [29,30]. Therefore, enhancing the interest of the population in health policies is a useful strategy for addressing moral challenges, albeit slightly.

This study aimed to determine the actual impact of the coverage of denture procedures for the elderly under NHI. We confirmed that denture treatment in the elderly increased, and therefore the policy had a positive impact on the older population. This impact was especially achieved by increasing the denture procedures of the low-income group and medical aid beneficiaries. Further investigations, especially those expanding the periods that were compared before and after the policy was implemented, would be helpful in overcoming the limitations of the existing study. Furthermore, the inclusion of multiple determinants of denture procedures is expedient.

The data used for this study are representative of the entire population because they are from numerous surveys conducted in Korea. This study had a positive result, because it confirmed that there was a slight change in denture procedure rates after the implementation of the policy in Korea. However, this study had some limitations related to the source of data for the study. We used KCHS data based on the responses of a randomized population to a survey in Korea. Because the survey population was not the entire population, the definition of study participants has limitations, such as selection bias or information bias. In addition, responses to the KCHS have potential bias because it is a self-reported survey. Nevertheless, the KCHS considers regional statistics certified by the country and assesses the reliability or validity of the survey every year to control the quality of the KCHS.

The government needs to increase the promotion of oral health examinations and dental health policies for the older population. In particular, the government should also provide and promote policies that relieve the financial burden of dental care for the elderly because the out-of-pocket expenditure for dental treatment is 84%, which is more than twice that for overall medical treatment [31]. Additionally, accurate medical information should be provided by professional health service providers because the elderly population depends on individuals such as doctors and medical staff to acquire medical information [32]. These policies would increase patient satisfaction with dental health and contribute to improving the quality of life of the elderly population.

## Figures and Tables

**Table 1:** Dental-health-related characteristics of study participants before and after coverage.

		65–74						≥75					
		Total	Having Denture		Not Having Denture		*p*	Total	Having Denture		Not Having Denture		*p*
			N	%	N	%			N	%	N	%	
Before coverage (2011)													
Total		4997	2319	46.4%	2678	53.6%		3507	1381	39.4%	2126	60.6%	
Missing teeth	Yes	1530	1091	71.3%	439	28.7%	<0.0001	1607	830	51.6%	777	48.4%	<0.0001
	No	3467	1228	35.4%	2239	64.6%		1900	551	29.0%	1349	71.0%	
Perceived oral health status	Good	96	51	53.1%	45	46.9%	0.0002	52	21	40.4%	31	59.6%	<0.0001
	Not bad	519	282	54.3%	237	45.7%		296	152	51.4%	144	48.6%	
	Bad	4382	1986	45.3%	2396	54.7%		3159	1208	38.2%	1951	61.8%	
Oral health examination	Yes	722	381	52.8%	341	47.2%	<0.0001	284	143	50.4%	141	49.6%	<0.0001
	No	4275	1938	45.3%	2337	54.7%		3223	1238	38.4%	1985	61.6%	
Periodontal disease	Yes	2688	1121	41.7%	1567	58.3%	<0.0001	1935	694	35.9%	1241	64.1%	<0.0001
	No	2274	1181	51.9%	1093	48.1%		1486	664	44.7%	822	55.3%	
	N/A	35	17	48.6%	18	51.4%		86	23	26.7%	63	73.3%	
After coverage (2013)													
Total		4875	2326	47.7%	2549	52.3%		3885	1683	43.3%	2202	56.7%	
Missing teeth	Yes	1433	1009	70.4%	424	29.6%	<0.0001	1596	911	57.1%	685	42.9%	<0.0001
	No	3442	1317	38.3%	2125	61.7%		2289	772	33.7%	1517	66.3%	
Perceived oral health status	Good	99	57	57.6%	42	42.4%	<0.0001	88	47	53.4%	41	46.6%	<0.0001
	Not bad	569	326	57.3%	243	42.7%		388	223	57.5%	165	42.5%	
	Bad	4207	1943	46.2%	2264	53.8%		3409	1413	41.4%	1996	58.6%	
Oral health examination	Yes	707	363	51.3%	344	48.7%	0.0009	368	182	49.5%	186	50.5%	<0.0001
	No	4168	1963	47.1%	2205	52.9%		3517	1501	42.7%	2016	57.3%	
Periodontal disease	Yes	2699	1198	44.4%	1501	55.6%	<0.0001	2068	838	40.5%	1230	59.5%	<0.0001
	No	2159	1123	52.0%	1036	48.0%		1799	841	46.7%	958	53.3%	
	N/A	17	5	29.4%	12	70.6%		18	4	22.2%	14	77.8%	

**Table 2:** Analysis of association between denture procedure and various characteristics.

Variables		OR	95% CI	
Age	65–74	1.000		
	≥75	0.875	0.859	0.890
Year	2011	1.000		
	2013	1.060	1.043	1.078
Age * Year		1.038	1.021	1.055
Missing teeth	Yes	1.969	1.934	2.004
	No	1.000		
Perceived oral health status	Good	1.000		
	Not bad	1.251	1.189	1.317
	Bad	0.727	0.696	0.760
Oral health examination	Yes	1.119	1.091	1.148
	No	1.000		
Periodontal disease	Yes	1.104	1.037	1.176
	No	1.000		
	N/A	0.618	0.547	0.699
Gender	M	0.751	0.730	0.773
	F	1.000		
Region	City	1.165	1.141	1.190
	Rural	1.000		
Economic activity	Yes	1.106	1.085	1.126
	No	1.000		
Family income	Q1 (low)	0.932	0.902	0.964
	Q2	1.024	0.991	1.058
	Q3	1.023	0.990	1.057
	Q4 (high)	1.000		
	N/A	0.994	0.940	1.050
Health insurance coverage	Medical aid	0.847	0.821	0.873
	NHI	1.000		
Education	≤Elementary	0.723	0.697	0.750
	≤Middle school	1.005	0.959	1.053
	≤high school	1.000		
	≥college	1.282	1.192	1.380
Marital status	Married	1.283	1.188	1.385
	Non-partnered	1.078	0.999	1.164
	Never married	1.000		
Smoking	Yes	1.034	1.008	1.061
	No	1.000		
Drinking	Yes	1.132	1.112	1.152
	No	1.000		

OR, Odds Ratio; 95% CI, 95% confidence interval; “Age * Year” means interaction term between the elderly individuals’ age and year.

**Table 3:** Difference-in differences (DID) analysis comparing target elderly individuals before and after coverage.

Variables			OR	95% CI	
Health insurance coverage					
Medical aid	Age	65–74	1.000		
		≥75	0.925	0.871	0.983
	Year	2011	1.000		
		2013	1.069	1.009	1.133
	Age * Year		1.107	1.045	1.172
NHI	Age	65–74	1.000		
		≥75	0.871	0.855	0.887
	Year	2011	1.000		
		2013	1.059	1.041	1.041
	Age * Year		1.033	1.015	1.051
Oral health examination					
Yes	Age	65–74	1.000		
		≥75	0.925	0.875	0.977
	Year	2011	1.000		
		2013	0.983	0.933	1.035
	Age * Year		1.048	0.995	1.104
No	Age	65–74	1.000		
		≥75	0.870	0.854	0.886
	Year	2011	1.000		
		2013	1.069	1.051	1.088
	Age * Year		1.035	1.017	1.053
Family income					
Q1 (Low)	Age	65–74	1.000		
		≥75	0.932	0.900	0.964
	Year	2011	1.000		
		2013	1.029	0.996	1.064
	Age * Year		1.074	1.039	1.110
Q2	Age	65–74	1.000		
		≥75	0.849	0.819	0.880
	Year	2011	1.000		
		2013	1.084	1.047	1.123
	Age * Year		1.037	1.002	1.074
Q3	Age	65–74	1.000		
		≥75	0.854	0.822	0.888
	Year	2011	1.000		
		2013	1.031	0.995	1.069
	Age * Year		1.053	1.016	1.092
Q4 (High)	Age	65–74	1.000		
		≥75	0.863	0.828	0.899
	Year	2011	1.000		
		2013	1.077	1.038	1.117
	Age * Year		1.021	0.984	1.059
N/A	Age	65–74	1.000		
		≥75	0.865	0.801	0.934
	Year	2011	1.000		
		2013	1.165	1.083	1.253
	Age * Year		0.916	0.852	0.985

OR, Odds Ratio; 95% CI, 95% confidence interval; “Age * Year” means interaction term between the elderly individuals’ age and year. Adjusted for all variables such as sex, region, economic activity, education, marital status, smoking, drinking, missing teeth, perceived oral health status, and periodontal disease.

## Data Availability

Publicly available datasets were analyzed in this study. The data presented in this study are openly available in: [https://chs.cdc.go.kr/chs/rawDta/rawDtaProvdMain.do (accessed on 10 January 2021)].

## Supplementary Materials

The following are available online at https://www.mdpi.com/1660-4601/18/5/2283/s1, Table S1: General characteristics of study participants before and after coverage.
