# Supplementary material for: Change in Denture Procedures after Implementation of National Health Insurance Coverage for the Elderly in Korea: A Nationwide Database
Source: Int J Environ Res Public Health. 2021 Feb 25;18(5):2283. doi: 10.3390/ijerph18052283 (PMC7967673; doi:10.3390/ijerph18052283)
Supplement: Supplementary file 1 [file ijerph-18-02283-s001.pdf]

Supplementary Materials

Table S1. General characteristics of study participants before and after coverage

		65-74						≥75					
		Total	Having denture		Not having denture		P	Total	Having denture		Not having denture		P
			N	%	N	%			N	%	N	%	
Before coverage(2011)													
Total		4,997	2,319	46.4%	2,678	53.6%		3,507	1,381	39.4%	2,126	60.6%	
Gender	Male	2,295	1,023	44.6%	1,272	55.4%	0.0474	1,320	499	37.8%	821	62.2%	0.7914
	Female	2,702	1,296	48.0%	1,406	52.0%		2,187	882	40.3%	1,305	59.7%	
Living	City	1,067	529	49.6%	538	50.4%	<.0001	567	257	45.3%	310	54.7%	<.0001
	Rural	3,930	1,790	45.5%	2,140	54.5%		2,940	1,124	38.2%	1,816	61.8%	
Economic activity	Yes	2,177	1,033	47.5%	1,144	52.5%	0.0310	375	333	88.8%	42	11.2%	0.0875
	No	2,820	1,286	45.6%	1,534	54.4%		2,748	1,048	38.1%	1,700	61.9%	
Family Income	Q1 (Low)	1,035	445	43.0%	590	57.0%	0.0238	1,131	424	37.5%	707	62.5%	0.2789
	Q2	1,171	532	45.4%	639	54.6%		780	300	38.5%	480	61.5%	
	Q3	1,328	646	48.6%	682	51.4%		633	260	41.1%	373	58.9%	
	Q4 (High)	1,067	521	48.8%	546	51.2%		623	264	42.4%	359	57.6%	
	N/A	396	175	44.2%	221	55.8%		340	133	39.1%	207	60.9%	
Health Insurance coverage	Medical aid	408	158	38.7%	250	61.3%	<.0001	417	135	32.4%	282	67.6%	0.0020
	NHI	4,589	2,161	47.1%	2,428	52.9%		3,090	1,246	40.3%	1,844	59.7%	
Education	≤Elementary	3,684	1,654	44.9%	2,030	55.1%	<.0001	2,969	1,108	37.3%	1,861	62.7%	<.0001
	≤Middle school	609	308	50.6%	301	49.4%		205	96	46.8%	109	53.2%	
	≤high school	568	284	50.0%	284	50.0%		237	123	51.9%	114	48.1%	
	≥college	136	73	53.7%	63	46.3%		96	54	56.3%	42	43.8%	
Marital status	Never married	29	10	34.5%	19	65.5%	0.0001	21	8	38.1%	13	61.9%	<.0001
	Married	3,435	1,623	47.2%	1,812	52.8%		1,590	655	41.2%	935	58.8%	
	Non-partnered	1,533	687	44.8%	846	55.2%		1,896	718	37.9%	1,178	62.1%	
Smoking	Yes	2,081	966	46.4%	1,115	53.6%	0.7559	1,269	467	36.8%	802	63.2%	0.3070
	No	2,916	1,353	46.4%	1,563	53.6%		2,238	914	40.8%	1,324	59.2%	
Drinking	Yes	2,284	1,085	47.5%	1,199	52.5%	0.6459	1,093	470	43.0%	623	57.0%	<.0001
	No	2,713	1,234	45.5%	1,479	54.5%		2,414	911	37.7%	1,503	62.3%	
After coverage(2013)													
Total		4,875	2,326	47.7%	2,549	52.3%		3,885	1,683	43.3%	2,202	56.7%	
Gender	M	2,201	1,009	45.8%	1,192	54.2%	0.6129	1,495	617	41.3%	878	58.7%	0.2244
	F	2,674	1,317	49.3%	1,357	50.7%		2,390	1,066	44.6%	1,324	55.4%	
Living	City	1,113	591	53.1%	522	46.9%	<.0001	689	328	47.6%	361	52.4%	0.0002

	Rural	3,762	1,735	46.1%	2,027	53.9%		3,196	1,355	42.4%	1,841	57.6%	
Economic activity	Yes	2,165	1,061	49.0%	1,104	51.0%	0.2439	897	424	47.3%	473	52.7%	0.0087
	No	2,710	1,265	46.7%	1,445	53.3%		2,988	1,259	42.1%	1,729	57.9%	
Family Income	Q1 (Low)	924	381	41.2%	543	58.8%		1,220	521	42.7%	699	57.3%	
	Q2	1,106	531	48.0%	575	52.0%		929	400	43.1%	529	56.9%	
	Q3	1,444	696	48.2%	748	51.8%	<.0001	753	334	44.4%	419	55.6%	0.8419
	Q4 (High)	1,223	621	50.8%	602	49.2%		831	367	44.2%	464	55.8%	
	N/A	178	97	54.5%	81	45.5%		152	61	40.1%	91	59.9%	
Health Insurance coverage	Medical aid	366	130	35.5%	236	64.5%	<.0001	365	142	38.9%	223	61.1%	0.0026
	NHI	4,509	2,196	48.7%	2,313	51.3%		3,520	1,541	43.8%	1,979	56.2%	
Education	≤Elementary	3,407	1,584	46.5%	1,823	53.5%		3,231	1,358	42.0%	1,873	58.0%	
	≤Middle school	669	339	50.7%	330	49.3%	0.0018	255	116	45.5%	139	54.5%	<.0001
	≤high school	607	297	48.9%	310	51.1%		302	158	52.3%	144	47.7%	
	≥college	192	106	55.2%	86	44.8%		97	51	52.6%	46	47.4%	
Marital status	Never married	37	11	29.7%	26	70.3%		14	6	42.9%	8	57.1%	
	Married	3,264	1,573	48.2%	1,691	51.8%	0.1016	1,831	815	44.5%	1,016	55.5%	0.0376
	Non-partnered	1,574	741	47.1%	833	52.9%		2,040	862	42.3%	1,178	57.7%	
Smoking	Yes	1,985	922	46.4%	1,063	53.6%	0.9633	1,440	601	41.7%	839	58.3%	0.4436
	No	2,890	1,404	48.6%	1,486	51.4%		2,447	1,082	44.2%	1,365	55.8%	
Drinking	Yes	2,193	1,104	50.3%	1,089	49.7%	<.0001	1,226	556	45.4%	670	54.6%	0.0002
	No	2,682	1,222	45.6%	1,460	54.4%		2,661	1,127	42.4%	1,534	57.6%	
